# Preface to special topic on quantum computing

**DOI:** 10.1093/nsr/nwz007

**Published:** 2019-02-14

**Authors:** Guang-Can Guo, Mingsheng Ying

**Affiliations:** 1 Key Laboratory of Quantum Information, Chinese Academy of Sciences, University of Science and Technology of China, China; 2 Synergetic Innovation Center of Quantum Information & Quantum Physics, University of Science and Technology of China, China; 3 Centre for Quantum Software and Information, University of Technology Sydney, Australia; 4 State Key Laboratory of Computer Science, Institute of Software, Chinese Academy of Sciences, China; 5 Department of Computer Science and Technology, Tsinghua University, China

At the beginning of the 1980s, quantum computing was born to noble parents—Quantum Mechanics and Computer Science, two of the greatest intellectual triumphs of the twentieth century—with Richard Feynman and David Deutsch among its midwives. Theoretical research over the last 40 years revealed that quantum computers can outperform current computers in a number of important computational tasks, particularly given the discovery of several celebrated quantum algorithms, such as Shor's for factoring, Grover's for searching, quantum simulation and HHL (Aram Harrow-Avinatan Hassidim-Seth Lloyd) for systems of linear equations. This research also foreshadows several promising application areas, including the simulation of quantum chemistry, security and privacy, and machine learning. With the expectation that quantum computers will be able to solve problems of significant economic and societal importance that are intractable by current computers, governments and industries around the globe have been heavily investing in building practical quantum computing hardware and software over the last 10 years. In particular, it is anticipated that ‘quantum supremacy' will be achieved by NISQ (Noisy Intermediate Scale Quantum) devices in the near future. At this pivotal time—the dawn of the quantum computing era—we invite leading experts to review several selected quantum computing topics, with a special hope that these articles will encourage new-generation researchers to enter this exciting field. These topics will by no means give a full picture of the field, however, due to the limited number of papers allowed in this special issue.

The Highlight article, ‘The complexity-theoretic Bell inequality' by Zhengfeng Ji, represents a recent significant advance in quantum complexity theory that contributes to our understanding of the separation between the classical and quantum worlds.

Does this separation imply the certain super-power of quantum systems? In the first Perspective article, Man-Hong Yung clarifies several fundamental concepts that will help the reader to better understand the term ‘quantum supremacy'—that is, the superiority of quantum computing over classical devices for a well-defined computational problem, and how it can be achieved.

After attaining ‘quantum supremacy', the next target should be practical quantum computing. The second Perspective article, by Mark Saffman, is an introduction to quantum computing with neutral atoms that reflects one of the main approaches to physically realizing large-scale quantum computing. The article also contributes a vision of this area in the next few years.

Once large-scalable and functional quantum computers are available, where and how we can use them? The Perspective article by Jonathan Allcock and Shengyu Zhang discusses several models of quantum machine learning—one of the most promising applications of quantum computing—with an emphasis on quantum neural networks.

How can an end-user without a good knowledge of quantum mechanics trust the outcome of a quantum computer? In the last Perspective article, Mingsheng Ying and Yuan Feng introduce model-checking techniques designed to verify both quantum circuits (hardware) and quantum programs (software).

A review on one of the approaches to *solid* state quantum computing is featured at the end of this special issue so that, when you finish reading the previous articles, you have a *solid* confidence that you will be able to use a quantum computer in the not too distant future. In this review, Hai-Ou Li and Guoping Guo offer an overview of semiconductor quantum computing, from the initialization, control and readout of qubits to the architecture of fault-tolerant quantum computers.

